# Novel *SCNN1A* gene splicing-site mutation causing autosomal recessive pseudohypoaldosteronism type 1 (PHA1) in two Italian patients belonging to the same small town

**DOI:** 10.1186/s13052-021-01080-x

**Published:** 2021-06-16

**Authors:** Gregorio Serra, Vincenzo Antona, Maria Michela D’Alessandro, Maria Cristina Maggio, Vincenzo Verde, Giovanni Corsello

**Affiliations:** 1 Department of Health Promotion, Mother and Child Care, Internal Medicine and Medical Specialties “G. D’Alessandro”, University of Palermo, Palermo, Italy; 2 Department of Pediatric Nephrology, Children’s Hospital “G. Di Cristina”, Palermo, Italy

**Keywords:** Pseudohypoaldosteronism, ENaC, *SCNN1A* gene, Splicing mutation, Next generation sequencing

## Abstract

**Introduction:**

Pseudohypoaldosteronism type 1 (PHA1) is a rare genetic disease due to the peripheral resistance to aldosterone. Its clinical spectrum includes neonatal salt loss syndrome with hyponatremia and hypochloraemia, hyperkalemia, metabolic acidosis and increased plasmatic levels of aldosterone. Two genetically distinct forms of disease, renal and systemic, have been described, showing a wide clinical expressivity. Mutations in the genes encoding for the subunits of the epithelial sodium channels (ENaC) are responsible for generalized PHA1.

**Patients’ presentation:**

We hereby report on two Italian patients with generalized PHA1, coming from the same small town in the center of Sicily. The first patient is a male child, born from the first pregnancy of healthy consanguineous Sicilian parents. A novel *SCNN1A* (sodium channel epithelial subunit alpha) gene mutation, inherited from both heterozygous parents, was identified by next generation sequencing (NGS) in the homozygous child (and later, also in the heterozygous maternal aunt). A more detailed family history disclosed a possible related twenty-year-old girl, belonging to the same Sicilian small town, with referred neonatal salt loss syndrome associated to hyperkalemia, and subsequent normal growth and neurodevelopment. This second patient had a PHA1 clinical diagnosis when she was about 1 year old. The genetic investigation was, then, extended to her and to her family, revealing the same mutation in the homozygous girl and in the heterozygous parents.

**Conclusions:**

The neonatologist should consider PHA1 diagnosis in newborns showing hyponatremia, hyperkalemia and metabolic acidosis, after the exclusion of a salting-loss form of adrenogenital syndrome. The increased plasmatic levels of aldosterone and aldosterone/renin ratio, associated to a poor response to steroid administration, confirmed the diagnosis in the first present patient. An accurate family history may be decisive to identify the clinical picture. A multidisciplinary approach and close follow-up evaluations are requested, in view of optimal management, adequate growth and development of patients. Next generation sequencing (NGS) techniques allowed the identification of the *SCNN1A* gene mutation either in both patients or in other heterozygous family members, enabling also primary prevention of disease. Our report may broaden the knowledge of the genetic and molecular bases of PHA1, improving its clinical characterization and providing useful indications for the treatment of patients. Clinical approach must be personalized, also in relation to long-term survival and potential multiorgan complications.

## Introduction

Pseudohypoaldosteronism type 1 (PHA1) is a rare genetic disease due to the peripheral resistance to aldosterone [1]. It was first reported by Cheek and Perry in 1958 [2]. Clinical spectrum with neonatal onset includes salt loss, hyponatremia, hypochloremia, hyperkalemia, metabolic acidosis and increased plasmatic levels of aldosterone [3]. Two forms of disease, renal (autosomal dominant, MIM#177735) and systemic (autosomal recessive, MIM#264350), have been described. They are genetically distinct, and show wide clinical expressivity. The most severe generalized PHA1 is caused by mutations in the genes encoding for the subunits of the epithelial sodium channels (ENaC) [3]. The alpha (*SCNN1A*, sodium channel epithelial subunit alpha) is mapped in 12p13, the beta (*SCNN1B*) in 16p12.2-p12.1, and the gamma (*SCNN1G*) in 16p12 [4]. Congenital adrenal hyperplasia (CAH) due to 21-β-hydroxylase or 3-β-hydroxysteroid dehydrogenase deficiency, hypoaldosteronism due to aldosterone deficiency, and Bartter syndrome should be considered as possible differential diagnosis in affected newborns [5, 6].

We hereby report on two Italian patients with generalized PHA1, coming from the same small town in the center of Sicily. Our report highlights how neonatologists should consider its diagnosis, after the exclusion of a salting-loss form of adrenogenital syndrome. We describe its rapid diagnostic suspicion, complex initial management, and the relevant role of next generation sequencing (NGS) techniques in defining the genetic profile of patients, as well as of carriers within families, also in view of primary prevention of disease.

## Patients’ presentation

A male newborn was delivered at term of the first uneventful pregnancy, by spontaneous vaginal delivery. Parents were healthy and consanguineous (grandparents first cousins), coming from a small town in the center of Sicily. Anthropometric measures at birth were: weight 4170 g(98th centile), length 55 cm (> 99th centile), head circumference 35.8 cm (86th centile). Apgar score was 10/10. He was exclusively breastfed, and postnatal clinical course was uneventful. Jaundice due to increased indirect bilirubin levels appeared on the third day of life, and requested phototherapy for 5 days. On the eighth day of life, progressive feeding difficulties, poor sucking, hyporeactivity and lethargy, tachycardia and profuse sweating were suddenly observed. He was transferred to the neonatal intensive care unit (NICU), where a severe salt loss syndrome with hyponatremia and hypochloremia (117 and 92 mEq/l, respectively), hyperkalaemia (9.6 mEq/l), and metabolic acidosis (pH 7.15, HCO_3_^−−^ 10.8 mmol/l, BE − 19.5 mmol/l) with normal anion gap were found. Urine analysis showed pH 5, while blood creatinine and urea nitrogen, as well as brain, heart and abdomen ultrasound examinations were normal, without revealing signs of hyperplasia of the adrenal glands and/or abnormalities of the kidneys and the urinary tract. Intravenous (IV) rehydration therapy with sodium chloride and sodium bicarbonate was started, for correction of hyponatremia and metabolic acidosis, respectively. Meanwhile, a treatment with fludrocortisone acetate (0.1 mg/day) and hydrocortisone (maximum dose 10 mg/m^2^/day) was also begun. Due to persistence of severe hyperkalemia, IV infusion of insulin and rectal, and subsequently oral, sodium polystyrene sulfate administration were performed. Breastfeeding was stopped, and the baby was fed with a special low-potassium formula. Hormonal parameters showed normal renin levels (2.1 ng/dl, normal values [n.v.] 0.25–3.58), significantly increased aldosterone ones (80.6 ng/dl, n.v. 0.37–3.1), aldosterone/renin ratio (38.37, n.v. < 20), 17-OH progesterone (> 16 ng/ml, n.v. 0.32–3.32), and low of ACTH (2.24 ng/l, n.v. 5–55) and cortisol (7.37 μg/dl, n.v. 6.2–19.4) (Table 1). Furthermore, high urinary levels of sodium (436 mEq/l, n.v. 54–150), and low of potassium (1 mEq/l, n.v. 20–80), in addition to parental consanguinity strengthened the diagnostic suspicion of PHA1. Then, NGS analysis of the genes involved in pseudohypoaldosteronism was performed. The *SCNN1A* gene mutation c.685-1G > A was identified in the homozygous proband, and in the heterozygous parents. Later, *SCNN1A* gene sequencing was performed also in the healthy maternal aunt, revealing the same heterozygous mutation.

**Table 1:** Serum and urinary electrolytes, and hormonal parameters of our first patient

	Admission to NICU (8 days)	Discharge (3 months)	Follow-up (6 months)
Na (mEq/l)	117	139	139
K (mEq/l)	9.6	4	3.91
Cl (mEq/l)	92	106	108.3
Urinary Na (mEq/l) (n.v. 54–150)	436		352
Urinary K (mEq/l) (n.v. 20–80)	1		3
ACTH (ng/l) (n.v. 5–55)	2.24	39.5	20
Aldosterone (ng/dl) (n.v. 0.37–3.1)	80.6	6.6	< 3.7
Renin (ng/dl) (n.v. 0.25–3.58)	2.1		< 1.8
Cortisol (μg/dl) (n.v. 6.2–19.4)	7.37		6.27
17-OH progesterone (ng/ml) (n.v. 0.32–3.32)	> 16	3.16	1.26

**Fig. 1:**
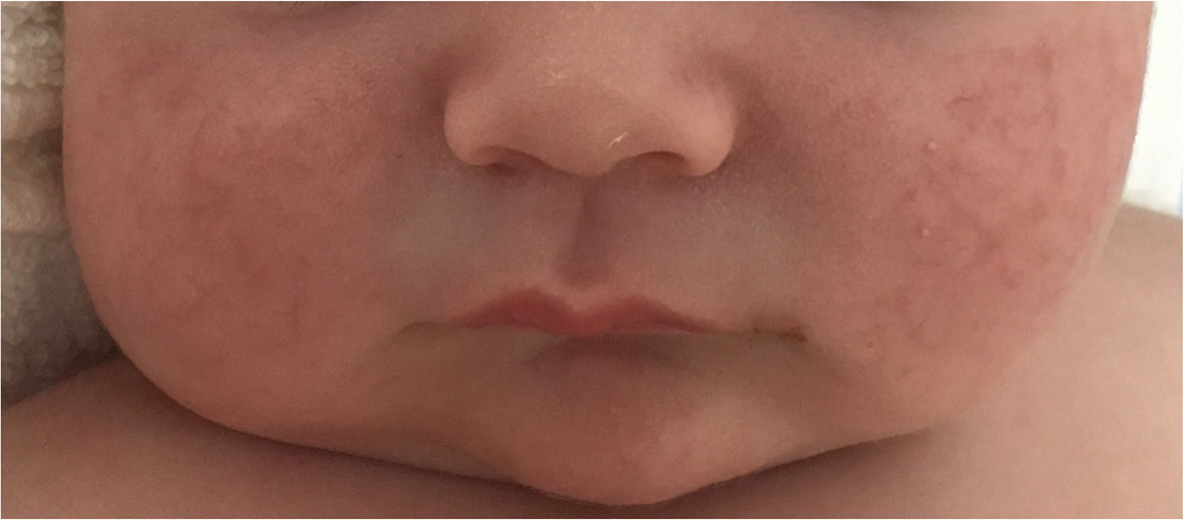
First patient: *miliaria rubra* on the face

**Fig. 2:**
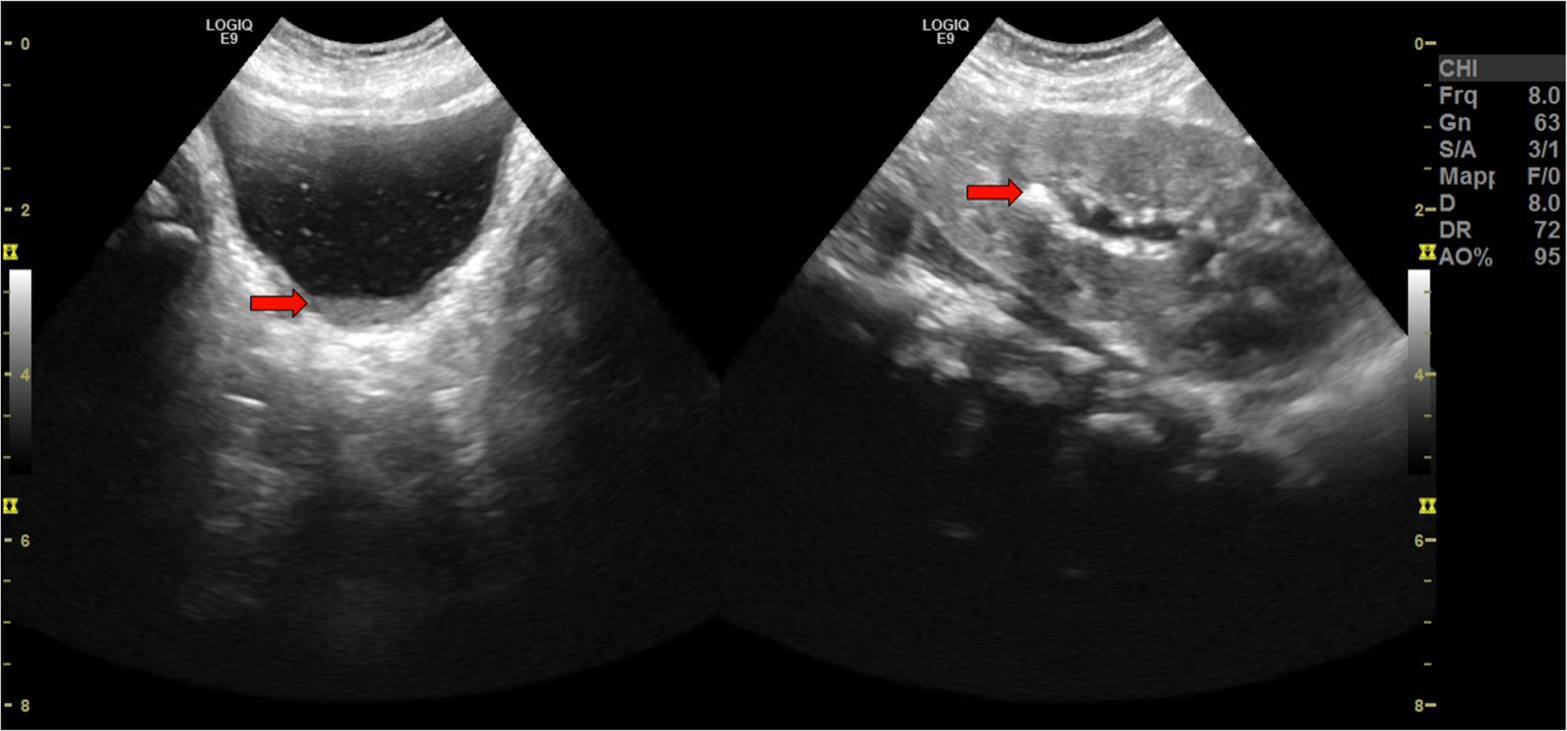
First patient’s renal and urinary tract ultrasound: small hyperechoic formations, compatible with calcium oxalate crystals, in the left kidney (the arrow indicates the largest, with maximum diameter of about 3 mm) and bladder (the arrow indicates their sediment)

The following clinical course of the newborn was characterized by *miliaria rubra* observed in the face (Fig. 1), without conjunctivitis and/or other signs of ocular involvement. At the age of 2 months, because of hypercalciuria, hyperoxaluria and crystals in the left kidney and bladder(Fig. 2), sodium citrate was associated in the treatment. Furthermore, the sweat test detected increased sodium levels (117 mEq/l, n.v. 15–65). He was discharged at 3 months of age in good general condition, adequate weight and length growth, neuromotor development and control of serum electrolytes and hormone levels (Table 1), due to treatment with fludrocortisone acetate (0.1 mg/day), sodium chloride (14 mEq/kg/day divided in 6 administrations), sodium citrate (250 mg/day in 2 divided doses) and sodium polystyrene sulfate (330 mg/kg/day in 2 divided doses), and temporary suspension of vitamin D supplementation.

The child currently is 6 months old, and shows adequate growth: weight 8.1 Kg (58th centile), length 72 cm (98th centile), and head circumference 42.5 cm (25th centile) (according to World Health Organization growth standards for neonatal and infant close monitoring) [7]. He is included in a multidisciplinary follow-up, and still treated with low-potassium formula, as well as oral fludrocortisone acetate (0.1 mg/day), sodium chloride (4.5 mEq/kg/day), sodium citrate (500 mg/day), and sodium polystyrene sulfate (300 mg/kg/day), with satisfying control of hormone levels and serum and urinary electrolytes including calcium, which enabled reintroduction of Vitamin D supplementation (Table 1).

Within a detailed medical history of the family, a possible related twenty-year-old girl belonging to the same Sicilian small town, with referred neonatal salt loss syndrome and hyperkalemia, failure to thrive in the first year of life and subsequent normal growth and neurodevelopment, was found. This second patient had a PHA1 clinical diagnosis when she was about 1 year old. Referring to a remote and unfocused coefficient of consanguinity, the genetic investigation was, then, extended to this further proband and to her family, allowing the identification of the same mutation in the homozygous girl and in the heterozygous parents. She currently is under treatment with low-potassium diet, sodium chloride and ion exchange resin, and has a normal life.

## Discussion and conclusions

PHA1 occurs in two forms, renal and systemic, genetically distinct and with wide clinical expressivity. Patients with generalized PHA1 are at risk of fatal salt loss episodes with severe hyperkalemia, from the neonatal age to adulthood [8]. Some of them may also have recurrent respiratory infections, mimicking cystic fibrosis [9]. Multiorgan involvement is due to sodium excretion in the distal nephron, in the sweat and salivary glands, and respiratory and intestinal mucosae [9]. In some patients, IV correction with sodium chloride and insulin administration may restore the Na/K balance. Ion exchange resins, and rarely dialysis, may be necessary [9]. Close monitoring of blood ion levels and frequent clinical evaluations are requested [10]. Skin lesions like *miliaria rubra*, for the high sweat sodium concentration, are a clinical sign of disease, and they were observed in our first patient [11]. Ocular involvement with enlarged meibomian glands is also described in few subjects [12]. The anamnestic data of parental consanguinity restricted in our newborn the diagnostic hypotheses to the systemic form of PHA1 [13, 14], which was confirmed by NGS analysis. The *SCNN1A* gene mutation identified in the proband, not previously reported in the literature, is located in a splicing site [15]. In vitro functional studies indicate a detrimental effect on the structure/function of the encoded protein, suggesting a pathogenic role of such genetic abnormality. This is supported by the identification of the same mutation in the homozygous possible related second patient, and in her heterozygous parents.

The progressive clinical improvement of our first patient, associated to gradual normalization of hormonal profile, depended on sodium chloride supplementation, steroid therapy, which gradually inhibits cortisol levels by stimulating the residual activity of mineralocorticoid receptors [16], and ion exchange resin, having a relevant role in the reduction of serum potassium [10]. Among the side effects of the latter, hypercalcemia and hypercalciuria are reported [17]. They were, indeed, observed in the first present patient, and treated with sodium citrate. Furthermore, the restriction of potassium intake may have had an additional therapeutic effect, as it increases the reabsorption of sodium through the Na^+^ Cl^−^ cotransporter (NCC), alternative to ENaC, in the distal nephron of affected subjects [8]. In PHA1 patients poor weight gain is described during the first 2 years of life [10]. Our infant has always shown an adequate growth velocity, also due to the caloric intake provided by the special formula which was administered. The salt-added formula may cause dislike, cough and vomit. However, it was well tolerated in our baby.

Few known mutations are associated with systemic PHA1. Most of them, including the one here described, involve the *SCNN1A* gene [18]. These are mainly deletions, insertions, or splicing mutations, which may cause abolition or severe malfunctioning of the encoded protein (subunit of ENaC) [1, 3, 19, 20]. Correlations between missense mutations and milder forms of disease are hypothesized. By converse, non-missense mutations (deletions, insertions and splicing) are observed in subjects with a more severe clinical picture, and this association is also present in our first patient [9, 18]. However, due to scarcity of in vitro functional studies, it is not possible to precisely define the effect of a specific mutation on structure and function of ENaC [18, 21]. Indeed, a genotype-phenotype relationship is not fully established to date, also considering the few cases described. Moreover, even in presence of non-missense mutations, clinical improvement over time is observed, which may be partly explained by the progressive increase in the caliber of the airways. This may be hypothesized also in our first patient, based on the favorable evolution observed in the second one. Therefore, other factors (epigenetic, environmental), may contribute to a less severe clinical course in some subjects [9, 22–24].

In newborns with hyponatremia, hyperkalaemia and metabolic acidosis, a PHA1 diagnosis should be considered, after the exclusion of a salting-loss form of adrenogenital syndrome. In addition, if normal kidney function tests and anion gap, urinary pH 5 and lack of response to furosemide are present, a type IV renal tubular acidosis is outlined, and the diagnostic suspicion may be further strengthened. In affected subjects increased plasmatic levels of aldosterone and aldosterone/renin ratio, associated with poor response to steroid administration are specific markers of disease. An accurate family history is often decisive for the diagnosis. The treatment relies on high dose sodium chloride, fludrocortisone and glucocorticoids, insulin, and ion exchange resins. It may be useful to reduce potassium intake with the use of special formulas. Clinicians must gradually balance the doses of drugs, based on electrolyte and hormone levels. Respiratory infections must be prevented by close follow-up evaluations, as well as careful and complete vaccination schedule, including anti-pneumococcal and anti-hemophilus b doses. Thus, a multidisciplinary team is needed for optimal management and adequate growth and development of these patients [25].

NGS techniques allowed the identification of *SCNN1A* gene mutations in present patients (homozygous), as well as in their parents and in the aunt of one of them (heterozygous). Indeed, detection of genetic variants in affected subjects and healthy carriers may be relevant for suggesting new elements of the genotype-phenotype correlations, and for a precise reproductive counselling, also in view of primary and/or secondary prevention of disease [20, 26, 27]. Our report may broaden the knowledge of the genetic and molecular bases of PHA1, improve its clinical characterization and provide useful indications for the treatment of patients. Clinical approach must be personalized, also in relation to long-term survival of affected subjects and potential multiorgan complications in children with unsatisfactory control of disease.

## Data Availability

The datasets used and analyzed during the current study are available from the corresponding author on reasonable request.

## Abbreviations

ACTH: Adrenocorticotropic hormone
BE: Base excess
CAH: Congenital adrenal hyperplasia
ENaC: Epithelial sodium channels
IV: Intravenous
NCC: Na^+^ Cl^−^ cotransporter
NGS: Next generation sequencing
NICU: Neonatal intensive care unit
n.v.: Normal values
PHA1: Pseudohypoaldosteronism type 1
*SCNN1A*: Sodium channel epithelial subunit alpha

## References

[1] Wang J, Yu T, Yin L, Li J, Yu L, Shen Y, Yu Y, Shen Y, Fu Q (2013). Novel mutations in the *SCNN1A* gene causing Pseudohypoaldosteronism type 1. PLoS One.

[2] Cheek DB, Perry JV (1958). A salt wasting syndrome in infancy. Arch Dis Child.

[3] Riepe FG (2009). Clinical and molecular features of type 1 pseudohypoaldosteronism. Horm Res.

[4] Furgeson SB, Linas S (2010). Mechanisms of type I and type II pseudohypoaldosteronism. J Am Soc Nephrol.

[5] Manikam L, Cornes MP, Kalra D, Ford C, Gama R (2011). Transient pseudohypoaldosteronism masquerading as congenital adrenal hyperplasia. Ann Clin Biochem.

[6] Schaedel C, Marthinsen L, Kristoffersson AC, Kornfӓlt R, Nilsson KO, Orlenius B, Holmberg L (1999). Lung symptoms in pseudohypoaldosteronism type 1 are associated with deficiency of the alpha-subunit of the epithelial sodium channel. J Pediatr.

[7] World Health Organization (2021). Child growth standards.

[8] Adachi M, Tajima T, Muroya K (2020). Dietary potassium restriction attenuates urinary sodium wasting in the generalized form of pseudohypoaldosteronism type 1. CEN Case Rep.

[9] Welzel M, Akin L, Büscher A, Güran T, Hauffa BP, Högler W, Leonards J, Karges B, Kentrup H, Kirel B, Senses EE, Tekin N, Holterhus PM, Riepe FG (2013). Five novel mutations in the *SCNN1A* gene causing autosomal recessive pseudohypoaldosteronism type 1. Eur J Endocrinol.

[10] Güran T, Değirmenci S, Bulut İK, Say A, Riepe FG, Güran Ö (2011). Critical points in the management of pseudohypoaldosteronism type 1. J Clin Res Pediatr Endocrinol.

[11] Eliwa MS, El-Emmawie AH, Saeed MA (2014). Ocular and skin manifestations in systemic pseudohypoaldosteronism. BMJ Case Rep.

[12] Nasir A, Abou NI (2012). Unique eyelid manifestations in type 1 pseudohypoaldosteronism. Arch Dis Child Fetal Neonatal Ed.

[13] Serra G, Corsello G, Antona V, D'Alessandro MM, Cassata N, Cimador M, Giuffrè M, Schierz IAM, Piro E (2020). Autosomal recessive polycystic kidney disease: case report of a newborn with rare *PKHD1* mutation, rapid renal enlargement and early fatal outcome. Ital J Pediatr.

[14] Piro E, Serra G, Schierz IAM, Giuffrè M, Corsello G (2020). Neonatal ten-year retrospective study on neural tube defects in a second level university hospital. Ital J Pediatr.

[15] Silva N, Costa M, Silva A, Sá C, Martins S, Antunes A, Marques O, Castedo S, Pereira A (2013). A case of systemic pseudohypoaldosteronism with a novel mutation in the *SCNN1A* gene. Endocrinol Nutr.

[16] Arai K, Tsigos C, Suzuki Y, Irony I, Karl M, Listwak S, Chrousos GP (1994). Physiological and molecular aspects of mineralocorticoid receptor action in pseudohypoaldosteronism: a responsiveness test and therapy. J Clin Endocrinol Metab.

[17] Shalev H, Ohali M, Abrason O (1994). Nephrocalcinosis in pseudohypoaldosteronism and the effect of indomethacin therapy. J Pediatr.

[18] Edelheit O, Hanukoglu I, Gizewska M, Kandemir N, Tenenbaum-Rakover Y, Yurdakök M, Zajaczek S, Hanukoglu A (2005). Novel mutations in epithelial sodium channel (ENaC) subunit genes and phenotypic expression of multisystem pseudohypoaldosteronism. Clin Endocrinol.

[19] Dogan CS, Erdem D, Mesut P, Merve A, Sema A, Iffet B, Afig B (2012). A novel splice site mutation of the beta subunit gene of epithelial sodium channel (ENaC) in one Turkish patient with a systemic form of pseudohypoaldosteronism type 1. J Pediatr Endocrinol Metab.

[20] Piro E, Schierz IAM, Antona V, Pappalardo MP, Giuffrè M, Serra G, Corsello G (2020). Neonatal hyperinsulinemic hypoglycemia: case report of kabuki syndrome due to a novel *KMT2D* splicing-site mutation. Ital J Pediatr.

[21] Hanukoglu I, Hanukoglu A (2016). Epithelial sodium channel (ENaC) family: phylogeny, structure-function, tissue distribution, and associated inherited diseases. Gene..

[22] Serra G, Antona V, Corsello G, Zara F, Piro E, Falsaperla R (2019). *NF1* microdeletion syndrome: case report of two new patients. Ital J Pediatr.

[23] Corsello G, Antona V, Serra G, Zara F, Giambrone C, Lagalla L, Piccione M, Piro E (2018). Clinical and molecular characterization of 112 single-center patients with Neurofibromatosis type 1. Ital J Pediatr.

[24] Serra G, Antona V, Schierz M, Vecchio D, Piro E, Corsello G (2018). Esophageal atresia and Beckwith–Wiedemann syndrome in one of the naturally conceived discordant newborn twins: first report. Clin Case Rep.

[25] Liotta A, Maggio MC, Iachininoto R, Bellipanni PF, Calì G, Arena V, Arena F (2004). Fetal pseudohypoaldosteronism: rare cause of hydramnios. Pediatr Med Chir.

[26] Borghesi A, Mencarelli MA, Memo L, Ferrero GB, Bartuli A, Genuardi M, Stronati M, Villani A, Renieri A, Corsello G, their respective Scientific Societies (2017). Intersociety policy statement on the use of whole-exome sequencing in the critically ill newborn infant. Ital J Pediatr.

[27] Piro E, Serra G, Antona V, Giuffrè M, Giorgio E, Sirchia F, Schierz IAM, Brusco A, Corsello G (2020). Novel LRPPRC compound heterozygous mutation in a child with early-onset Leigh syndrome French-Canadian type: case report of an Italian patient. Ital J Pediatr.

